# Physiology and transcriptome analysis of the response mechanism of *Solidago canadensis* to the nitrogen addition environment

**DOI:** 10.3389/fpls.2023.1005023

**Published:** 2023-02-14

**Authors:** Miao Wu, Huiyuan Liu, Ying Zhang, Bingbing Li, Tao Zhu, Man Sun

**Affiliations:** School of Life Science and Engineering, Henan University of Urban Construction, Pingdingshan, Henan, China

**Keywords:** plant invasion, nitrogen deposition, *Solidago canadensis*, gene expression, physiological analysis

## Abstract

*Solidago canadensis* is an invasive plant that can adapt to variable environmental conditions. To explore the molecular mechanism of the response to nitrogen (N) addition conditions in *S. canadensis*, physiology and transcriptome analysis were performed with samples that cultured by natural and three N level conditions. Comparative analysis detected many differentially expressed genes (DEGs), including the function of plant growth and development, photosynthesis, antioxidant, sugar metabolism and secondary metabolism pathways. Most genes encoding proteins involved in plant growth, circadian rhythm and photosynthesis were upregulated. Furthermore, secondary metabolism-related genes were specifically expressed among the different groups; for example, most DEGs related to phenol and flavonoid synthesis were downregulated in the N-level environment. Most DEGs related to diterpenoid and monoterpenoid biosynthesis were upregulated. In addition, many physiological responses, such as antioxidant enzyme activities and chlorophyll and soluble sugar contents, were elevated by the N environment, which was consistent with the gene expression levels in each group. Collectively, our observations indicated that *S. canadensis* may be promoted by N deposition conditions with the alteration of plant growth, secondary metabolism and physiological accumulation.

## Introduction

Biological invasion is a global phenomenon and a threat to global biodiversity and the global economy (Simberloff et al., 2013; Diagne et al., 2021), representing a significant element of global change. Numerous studies have shown that plant invasion can reduce native plant performance. Therefore, invasive species will threaten global biodiversity, and experts are required to effectively determine the main causes of biological invasion (Pysek et al., 2012; Hui et al., 2021). The ecological and economic impact of species invasions showed that managers urgently need to provide meaningful suggestions for how to prevent invasions and how to prioritize the management of invasive species (Luo et al., 2020). To understand the impact of environmental changes caused by human activities on biological invasion, many studies have been performed to explore the responses of invasive plants in common environments (Huang et al., 2022).

The invasiveness of species or habitat conditions can be changed through environmental changes, which may facilitate or inhibit alien invaders (Nguyen et al., 2016; Merow et al., 2017). Global environmental change from human activities acts as the main factor influencing plant invasion in general (Bradley et al., 2010; Carboni et al., 2018; Questad et al., 2021). The main reason for the successful invasion of alien plants is related to the environment in which they grow (Heshmati et al., 2019). Thus, plant invasion can be significantly affected by environmental factors in the receptor habitat, affecting social stability and sustainable development (Ren et al., 2020). Human-mediated interference may increase the invasiveness of alien plants, such as nutrient fluctuations in nitrogen (N) and phosphorus (Zhang et al., 2022). Recently, the distribution of anthropogenic N, which is usually caused by social and urban development, rapid agricultural intensification, and increased fuel utilization, has rapidly increased global N deposition and significantly impacts ecosystems (Yu et al., 2019).

Previous studies have proven that N deposition is accelerated by profound effects on both natural and anthropogenic ecosystems (Yu et al., 2019). Recent research and analysis of the changes in N deposition in China have increased by approximately 60%, which is the highest value in the world (Dentener et al., 2006; Liu et al., 2013). Many studies have shown that N plays an essential role in the rapid growth of invasive plants (Parepa et al., 2013; Ribeiro et al., 2017; Luo et al., 2019). Therefore, N enrichment has become an increasingly important element in global environmental change and has been widely focused on its effect on plant biodiversity and invasive plant ranges (Payne et al., 2017; Midolo et al., 2019). Although some studies have implied that high N levels in soil will promote invasive species to develop into a peak or drop (Mattingly and Reynolds, 2014; Nguyen et al., 2016), there are some evidences that suggests that N addition and enrichment will increase the opportunity to form invasive species and finally facilitate plant flourishing into invasion (Bradley et al., 2010; Vallano et al., 2012; Seabloom et al., 2015; Liu et al., 2017; Liu et al., 2019). To better understand the mechanism of how global climate change affects the mechanism of plant invasion, many novel studies are being conducted. With the development of biology, next-generation RNA sequencing technologies (RNA-seq) have supplied an approach to describe the whole genome or transcriptomes and are being used to explore the regulatory mechanisms of plant responses to many conditions in which they grow (Chu et al., 2019; Khan et al., 2022). Therefore, transcriptomic profiling can be used as an emerging method to reveal the molecular mechanism of plants in response to different environments (Asim et al., 2021; Kayihan et al., 2021; Qu et al., 2021; Li et al., 2022).

*Solidago canadensis* L., native to North America, is an aggressive invasive plant that successfully invaded Europe, Australia, Asia and New Zealand (Dong et al., 2006; Szymura and Szymura, 2013; Nolf et al., 2014; Gusev, 2015; Dong et al., 2017; Ye et al., 2019). The species can colonize different growth environments and exhibit a variety of phenotypes to adapt to these environments (Li et al., 2016; Jin et al., 2020). Furthermore, recent studies have attempted to suggest that N deposition and enrichment may facilitate *S. canadensis* invasion and rapid adaptation (Peng et al., 2019; Wan et al., 2019). Extended studies will provide a meaningful model for exploring invasive plants in response to the climate change environment.

Some studies have proved that invasive plants may have corresponding changes in physiological, biochemical and related gene expression levels for the environmental changes (Chuang et al., 2022; Cocozza et al., 2022; Liu Z et al., 2022). Invasive plants may be affected their enzyme activities and the synthesis process of secondary metabolites for the altered external environment (Cocozza et al., 2022; Hura et al., 2022). In this study, to explore the response of *S. canadensis* to the N addition climate change in physiology and related gene expression, we conducted a simulated N addition environment to detect the effects of altered levels on the physiology and gene expression of *S. canadensis*. Our aims were as follows: (i) explore physiological differences in plants among different levels of N addition; (ii) screen the differentially expressed transcripts; and (iii) determine the mechanism by which N addition may enhance the invasive ability of *S. canadensis*.

## Materials and methods

### Plant materials and experimental design

*S. canadensis*, as an ornamental plant, was introduced into China in 1935. At present, it is distributed in many provinces of China (Oduor et al., 2022). Mature seeds were collected from a suburb of Wuhan city (30°32′N, 114°25′E) and sown into plastic pots with a diameter of 25 cm.

To simulate increasing rates of N deposition, a total of 12 samples from four differential levels of treatments with three independent replicates for each treatment were carried out. The control group was established by pure water, and the other three levels of simulated N deposition were added by a polyethylene injector with prepared NH_4_NO_3_ solution, which included low N with a deposition rate of 5 g m^−2^ yr^−1^ (N5), intermediate N with 10 g m^−2^ yr^−1^ (N10), and high N with 15 g m^−2^ yr^−1^ (N15). All treatments were applied for 5 weeks, and N solution was applied to the corresponding pots. In addition, to provide daily basic water needs, 500 ml of pure water was added every two days in each pot.

### Physiological measurements

The soluble sugars, MDA, chlorophyll, superoxide dismutase (SOD), catalase (CAT), and peroxidase (POD) were quantified. Each measurement was performed with three biological replicates. The chlorophyll content, which included total chlorophyll (Chl a+b) was detected by Wu et al. (2020b).

The content of soluble sugars was detected according to Quinet et al. (2012) with some modifications. The frozen samples (1 g) were put into liquid N, ground to powder, and then incubated at room temperature with 10 mL of pure water. The mixture solution was transferred into a centrifuge tube and centrifuged at 7000 rpm for 10 minutes. The supernatant was collected for estimation by anthrone reagent.

The measurements of malonaldehyde (MDA) and antioxidant enzyme activities were performed based on Cao et al. (2017) with minor modification. The powder of each sample was collected at 0.1 g to prepare 10 mL of 10% TCA solution and then centrifuged at 7000 rpm for 10 minutes. The supernatant (2 mL) was added to 2 mL of 0.6% thiobarbituric acid (TBA). The samples were heated in boiling water for 15 minutes and cooled immediately. The mixture solution was centrifuged at 7000 rpm for 10 minutes, and then the absorbance values were detected at 532 nm, 600 nm and 450 nm.

Antioxidant enzyme activities were assayed according to Zhong et al. (2017). Fresh leaf powder (0.1 g) was collected and incubated in phosphate buffer solution, which was precooled at 4 °C. The mixture was homogenized and then centrifuged at 7000 rpm at 4°C for 15 minutes. We collected the supernatant to detect the activity of SOD, CAT and POD.

### RNA and cDNA library preparation

TRIzol reagent (Invitrogen, Carlsbad, CA, USA) was used to isolate total RNA according to the protocol’s instructions, and the quantification of isolated RNA was detected by using a NanoDrop spectrophotometer (Thermo Fisher Scientific, Inc.). The quality and integrity of the RNA samples were assessed with a Bioanalyzer 2100 (Agilent, CA, USA). The total RNA of each sample that satisfied the range of OD260/280 values between 1.8 and 2.2 and an RNA integrity number (RIN) above 7.5 was used for cDNA library preparation. We collected 1 μg of RNA per sample and used it to construct a paired-end cDNA library, and each library was sequenced by the Illumina NovaSeq 6000 sequencing platform at Biomarker Technology (Beijing, China). All the clean data of this study were deposited in the National Genomics Data Center (https://ngdc.cncb.ac.cn/) and are accessible through BioProject accession number PRJCA010244.

### *De novo* transcriptome assembly and annotation

Trinity software (v2.5.1) was used to assemble transcripts (Grabherr et al., 2011), which satisfied the assumption that the assembly length should not be less than 200 bp. The redundant sequences were removed by cd-hit (v4.6.1) and clustered with Tgicl (v2.1) to merge the sequences with a similarity over 90%. The functions of the assembled unigenes were identified by searching against public databases, such as the nonredundant protein (Nr), Kyoto Encyclopedia of Genes and Genomes (KEGG), Gene Ontology (GO), and eggNOG databases, with a significance threshold of E-value ≤ 10^−5^.

### Gene expression pattern analysis

The assembled unigenes were used to map clean reads by Bowtie software, and the gene expression level was analysed and represented by using RSEM software based on fragments per kilobase of transcript sequence per million base pairs (FPKM) (Li and Dewey, 2011). The differentially expressed genes (DEGs) in each comparison group were identified by DESeq2, and the significant DEGs were collected based on a q-value < 0.01 and a fold change value ≥ 2. To explore which DEGs may play an important role in each comparison group, GO and KEGG pathway enrichment analyses of the DEGs were applied by the topGO R packages and KOBAS software, respectively.

### Gene coexpression network analysis

The weighted gene correlation network analysis (WGCNA) approach is usually performed to identify gene coexpression networks, which can be used to thoroughly investigate the correlation patterns across multiple samples. All DEGs between the different N level treatments and the control were applied with the R package WGCNA.

### Statistical analysis

The evaluation of statistical data, which observed under different N level treatment groups, was tested by normality (Shapiro-Wilk) and homogeneity (Bartlett). We would transform and normalize the data that need to reduce the heterogeneity of variance. ANOVAs were performed to evaluate the significant differences of physiological changes in *S. canadensis* under different nitrogen levels. *Post hoc* LSD tests were used to separate differences between pairs of treatments. Three biological replicates were collected and analysed by using variance, and the mean ( ± SD) with the significant difference analysis was satisfied a p value < 0.05.

### Validation of qRT-PCR analysis

The isolated RNA was reverse transcribed into cDNA by an M-MuLV cDNA Synthesis Kit (Sangon Biotech). The primers of this study for qRT-PCR were designed by Primer Premier 5.0 software to verify the relative expression level of randomly selected genes with the 18S gene as an internal control. The relative expression of these randomly selected genes was calculated with the 2^−△△Ct^ method. The PCR conditions were set as follows: predenaturation at 95°C for 10 min and 40 cycles of 95°C for 10 s and 60°C for 30 s (Wu et al., 2021).

## Result

### Physiological and biochemical responses to different N level environments

**Figure 1:**
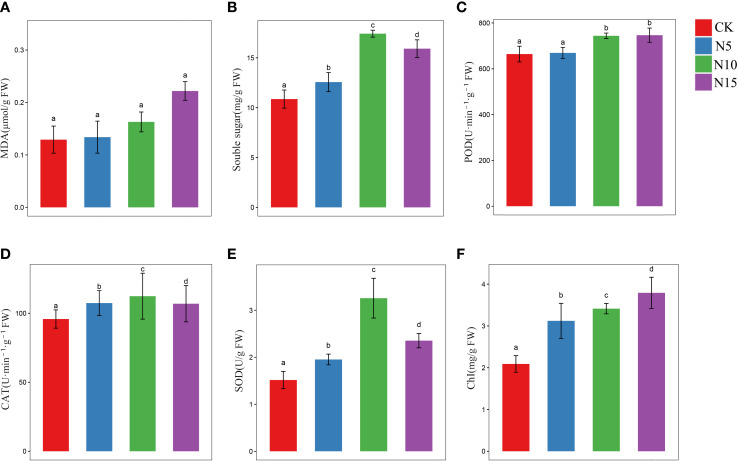
Physiological evaluate of *S. canadensis* in different N addition conditions. **(A, B, F)** The content of MDA, soluble sugar and total chlorophyll in leaves of various N level environment. **(C–E)** The activities of POD, SOD and CAT enzymes. The color bar represented each group (mean ± SE), and the significant differences among different N level groups was marked by letters (p <.05). The red, blue, green, and purple color bar represents CK, N5, N10, and N15 group, respectively.

The physiological and biochemical response analysis showed accumulation of proline, MDA and soluble sugar among different N level environments in *S. canadensis*. The levels of MDA increased with increasing N levels, while no significant changes were detected among the groups (**Figure 1A**). The result may show little harm to the cell membrane in *S*. *canadensis*. In addition, the soluble sugar contents reached a maximum in the N10 environment with significant changes (**Figure 1B**). This result showed a significant effect of energy metabolism in *S*. *canadensis* among different N level environments.

The detected antioxidant enzymes (POD, CAT and SOD) at the different N levels are depicted in **Figures 1C–E**. The activity of POD was increased in the N environment groups. No significant effect was found between the CK and N5 groups, and a significant increase was observed between the CK and N10 and N15 groups (**Figure 1C**). The activities of CAT and SOD reached a maximum in the N10 groups, with significant differences among the groups (**Figures 1D, E**).

The contents of photosynthetic pigment (total chlorophyll) in each group were all affected by the N environment, and a significant difference was also detected among the CK with N5, N10, and N15 groups (**Figure 1F**). This result indicates that photosynthesis may be promoted by the N environment.

### Overview of gene expression patterns

**Figure 2:**
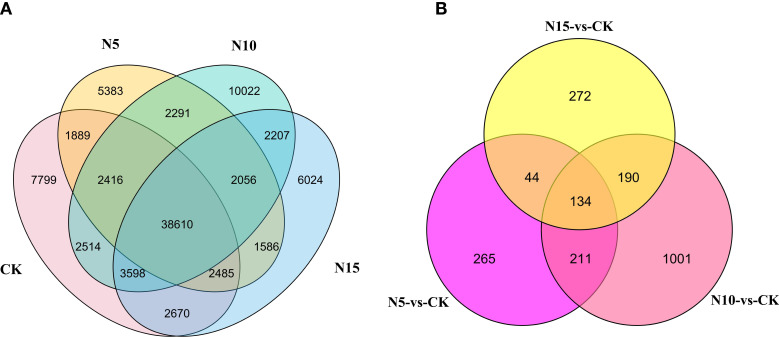
The overlap of expressed genes in each group and DEGs among three comparison groups. **(A)** The overlap of expressed genes in the CK, N5, N10, and N15 group. **(B)** The overlap of DEGs at CK-vs-N5, CK-vs-N10 and CK-vs-N15 comparison group.

We performed transcriptome analysis to explore the molecular changes in *S. canadensis* exposed to different doses of N. Fresh leaf samples with different N level conditions and one control check were used for RNA-seq analysis. Based on the deep sequencing of RNA-seq, 96.50 Gb clean reads with no less than 7 Gb per sample was obtained. A total of 106,423 unigenes were obtained according to the assembly result. As a result of the expression analysis, 91,550 unigenes were detected by FPKMs, with 61,981, 56,716, 63,714, and 59,236 unigenes in the CK, N5, N10, and N15 groups, respectively. Among these expressed genes in each group, 38,610 genes were detected in each of the four experimental groups, and 7,799, 5,383, 10,022 and 6,024 genes were specifically expressed in the CK, N5, N10 and N15 groups, respectively (**Figure 2A**).

### Analysis of differentially expressed genes

To better determine the difference in response to various N level conditions in *S. canadensis* leaves, three comparison groups were constructed, including N5-vs.-CK, N10-vs.-CK, and N15-vs.-CK. There were 654 DEGs in the comparison of N5-vs.-CK, with 372 being upregulated and 282 downregulated (**Table 1**). Furthermore, in the N10-vs.-CK comparison, 1,536 DEGs (745 upregulated and 791 downregulated) (**Table 1**) were detected. Under the N15 condition compared with the control group, we detected 640 DEGs (331 upregulated and 309 downregulated) (**Table 1**). It is possible that with the addition of N in the environment, some genes may be depressed. The overlap of DEGs showed that 134 DEGs were detected in all three groups; furthermore, 265 DEGs were specifically expressed in the N5-vs.-CK group, 1,001 in N10-vs.-CK, and 272 in N15-vs.-CK (**Figure 2B**).

**Table 1:** The number of DEGs in three comparison groups.

Groups	Total number	Up-regulated	Down-regulated
CK-vs-N5	654	372	282
CK-vs-N10	1536	745	791
CK-vs-N15	640	331	309

### GO functional analysis of DEGs

**Figure 3:**
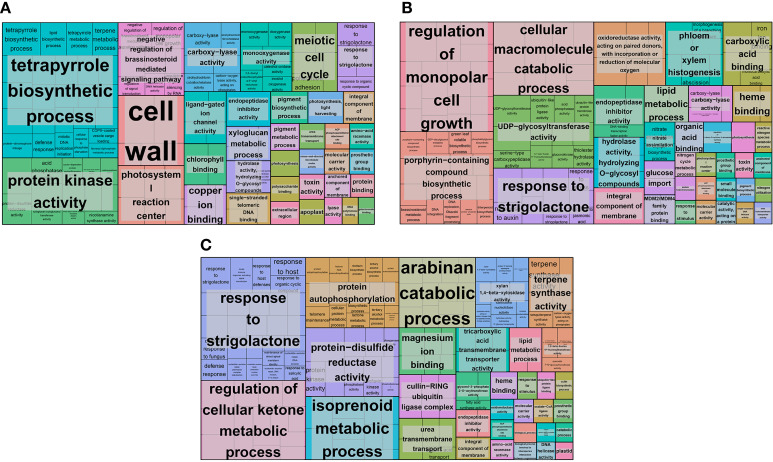
The distribution of DEGs and GO functional enrichment analysis. **(A)** The GO enrichment of CK-vs-N5 comparison; **(B)** The GO enrichment of CK-vs-N10 comparison. **(C)** The GO enrichment of CK-vs-N15 comparison. The enriched GO terms were presented in rectangles.

To identify and classify the functions of the DEGs, we performed GO classification analysis. In the N5-vs.-CK group, 471 DEGs were classified into 56 GO terms with three main categories: cellular component, molecular function and biological process. In the cellular component category, most DEGs were classified into the ‘membrane’, ‘cell’, ‘cell part’ and ‘membrane part’ subcategories (**Supplementary Table S1**). Most DEGs were classified into ‘catalytic activity’ and ‘binding’ in the molecular function category (**Supplementary Table S1**). In the biological process category, most DEGs were classified into ‘metabolic process’, ‘cellular process’ and ‘single-organism process’ (**Supplementary Table S1**). In addition, we performed GO functional enrichment analysis to identify insight genes that may be vital for each comparison group, and the significantly enriched GO terms were further identified by REVIGO. Many GO terms with biological functions, such as ‘tetrapyrrole biosynthetic process’, ‘photosystem I reaction center’, ‘negative regulation of brassinosteroid mediated signalling pathway’, ‘monooxygenase activity’, ‘chlorophyll binding’, ‘urea transmembrane xyloglucan transport’, and ‘photosynthesis’ (**Figure 3A**). These significantly enriched genes might play an essential functional role in the N5-vs.-CK group. In the N10-vs.-CK group, 1067 DEGs were classified into 46 GO terms of three categories. Most DEGs were classified into subcategories similar to those in the N5-vs.-CK group (**Supplementary Table S2**). The GO enrichment and REVIGO analysis found that except for some enriched GO items that were similar to the N5-vs.-CK group, many other items were also enriched, such as ‘regulation of monopolar cell growth’, ‘nitrate assimilation’, ‘nitrogen cycle metabolic process’ and ‘nitrogen utilization’ (**Figure 3B**). In the N15-vs.-CK comparison, 449 DEGs were classified into 39 GO terms. Most DEGs were classified into subcategories similar to the other two comparison groups (**Supplementary Table S3**). According to the GO enrichment and REVIGO analysis, some enriched GO terms were the same as those in the other two comparison groups, while some specifically enriched GO terms were also collected, such as ‘protein autophosphorylation’, ‘isoprenoid metabolic process’ and ‘terpene synthase activity’ (**Figure 3C**).

### KEGG pathway analysis of DEGs

**Figure 4:**
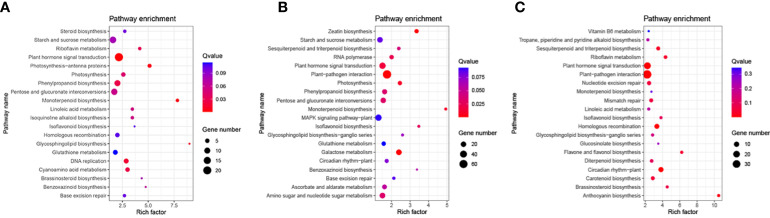
KEGG pathway functional enrichment analysis of DEGs. **(A)** The CK-vs-N5 comparison; **(B)** The CK-vs-N10 comparison. **(C)** The CK-vs-N15 comparison. The KEGG pathway name were presented in Y-axis, and the rich factor were presented in X-axis. The color bar and bubble size represent the corresponding q-value and number of genes respectively.

Based on the KEGG pathway enrichment analysis, 96 pathways were enriched DEGs in the N5-vs.-CK comparison group, which included ‘plant hormone signal transduction’, ‘starch and sucrose metabolism’ and ‘phenylpropanoid biosynthesis’ (**Supplementary Table S4**). To explore significant enrichment, the pathways were ranked by collected Q-values, which included ‘phenylpropanoid biosynthesis’, ‘monoterpenoid biosynthesis’, ‘plant hormone signal transduction’, ‘photosynthesis-antenna proteins’ and ‘brassinosteroid biosynthesis’ (**Figure 4A**). The significantly enriched DEGs in these pathways may be essential to respond to N5 conditions. In the N10-vs.-CK comparison group, DEGs were mapped into 106 pathways (**Supplementary Table S5**), and most of the DEGs were distributed in pathways similar to those in N5-vs.-CK. Based on the significant enrichment analysis, some of the collected pathways of the top 20 were the same as those in N5-vs.-CK, while some other pathways were also enriched, such as the ‘zeatin biosynthesis’, ‘sesquiterpenoid and triterpenoid biosynthesis’, ‘circadian rhythm-plant’, ‘MAPK signalling pathway’ and ‘glutathione metabolism’ pathways (**Figure 4B**). In the N15-vs.-CK comparison group, DEGs were mapped into 89 pathways (**Supplementary Table S6**), and most of the DEGs distributed similar pathways with the other two comparison groups. Some of the top 20 significant enriched pathways in this comparison group were same as N5-vs.-CK group, such as ‘riboflavin metabolism’, ‘linoleic acid metabolism’ and ‘brassinosteroid biosynthesis’, and some of the top 20 enriched pathways were same as N10-vs.-CK group, such as ‘plant-pathogen interaction’, ‘sesquiterpenoid and triterpenoid biosynthesis’ and ‘circadian rhythm-plant’. Three pathways of the top 20 were all collected in each comparison group that included pathways of ‘plant hormone signal transduction’, ‘isoflavonoid biosynthesis’ and ‘starch and sucrose metabolism’. Other pathways, such as ‘anthocyanin biosynthesis’ and ‘carotenoid biosynthesis’, were specifically enriched (**Figure 4C**). DEGs enriched in these pathways may be important in the response to N10 and N15 conditions. Thus, we inferred that with increasing N levels, the expressed functional genes may have changed.

### Plant growth and development related DEGs

**Figure 5:**
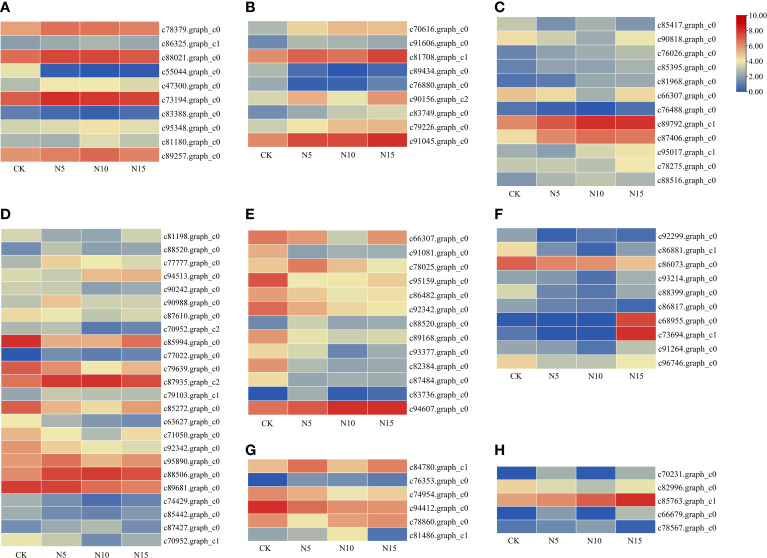
The expression profile of collected functional genes in various N level environments. **(A)** Photosynthesis; **(B)** Auxin; **(C)** Circadian rhythm; **(D)** Phenylpropanoid biosynthesis; **(E)** Flavonoids synthesis; **(F)** sesquiterpenoid and triterpenoid biosynthesis; **(G)** diterpenoid biosynthesis; **(H)** monoterpenoid biosynthesis.

According to the pathway functional enrichment analysis in the three comparison groups, a total of 10 DEGs were involved in the photosynthesis pathway. In the N5-vs.-CK comparison group, 6 DEGs were collected, with 5 upregulated and 1 downregulated DEGs, and in the N10-vs.-CK comparison group, 10 DEGs were collected, with 8 upregulated and 2 downregulated DEGs. In the N15-vs.-CK comparison group, one downregulated DEG was detected (**Figure 5A**). This result showed that with increasing N addition level, the expression and number of photosynthesis-related DEGs first increased and then decreased. Furthermore, 9 auxin-related DEGs were collected in the three comparison groups. In the N5-vs.-CK comparison group, 5 DEGs were detected, with 3 upregulated and 2 downregulated. Furthermore, 7 DEGs were collected in the N10-vs.-CK and N15-vs.-CK comparison groups, with 5 upregulated and 2 downregulated DEGs in the N10-vs.-CK comparison and 6 upregulated and 1 downregulated DEGs in the N15-vs.-CK comparison (**Figure 5B**). In addition, 12 DEGs were detected in the ‘circadian rhythm-plant’ pathway in the three comparison groups. In the N5-vs.-CK comparison group, 2 *CO* genes were detected and upregulated. In the N10-vs.-CK comparison group, 8 DEGs with 5 upregulated and 3 downregulated genes were detected, which included 2 *LHY*, 3 *CO*, 1 *TOC1*, 2 *COP1* and 1 *CHS* genes. In the N15-vs.-CK comparison group, 9 DEGs, including 8 upregulated and 1 downregulated DEGs, were detected, and except for most of the DEGs in the N10-vs.-CK comparison group, 3 *PIF3* genes were also detected (**Figure 5C**). These DEGs may play important functions in responding to different N level environments and regulating plant growth and development in *S. canadensis*.

### Secondary metabolism related DEGs

In this study, most secondary metabolism-related DEGs were collected and enriched in related pathways. For example, in the ‘phenylpropanoid biosynthesis’ pathway, 10 DEGs were collected, with 8 upregulated, such as the *F6H* and *HCT* genes, and 2 downregulated in the N5-vs.-CK comparison group. In the N10-vs.-CK comparison group, 17 DEGs, 12 downregulated, including the *F5H*, *HCT*, cinnamyl-alcohol dehydrogenase (*CAD*) and *CCR* genes, and 5 upregulated. In the N15-vs.-CK comparison group, most DEGs were also downregulated, such as the *PAL*, *CAD* and *HCT* genes (**Figure 5D**). Furthermore, in the flavonoid biosynthesis pathway, most DEGs were downregulated in the three comparison groups, including *CHS*, *CHI* and *FLS* genes (**Figure 5E**). Phenol and flavonoid synthesis may be depressed in *S. canadensis* in an elevated N level environment. Terpenoid biosynthesis-related DEGs, such as those in the ‘monoterpenoid biosynthesis’ and ‘diterpenoid biosynthesis’ pathways, were collected in the three comparison groups. In the comparison groups of N5-vs.-CK and N10-vs.-CK, all of the detected sesquiterpenoid and triterpenoid biosynthesis-related genes were downregulated, such as *GERD*, beta-amyrin synthase (*β-AS*) and *CYP* genes. Nevertheless, in the N15-vs.-CK comparison group, 2 *GERD* genes were upregulated (**Figure 5F**). In addition, most of the diterpenoid and monoterpenoid biosynthesis-related DEGs, such as kaurene synthase (*KS*), momilactone synthase (*MS*) and carboxylinalool synthase-related genes, were upregulated in the three comparison groups (**Figures 5G, H**). These results provided different regulation patterns of terpenoid synthesis in response to changes in the N environment.

### Gene expression network analysis

**Figure 6:**
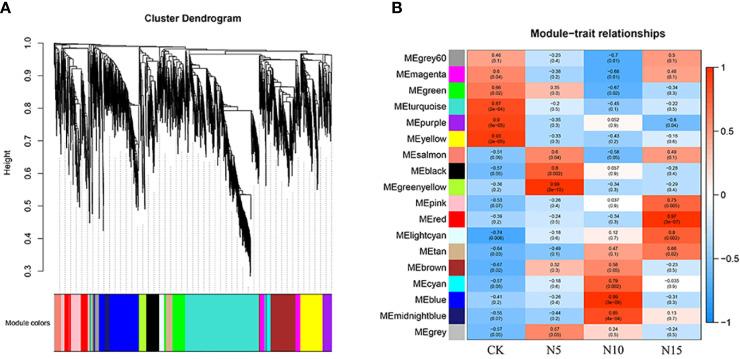
The analysis of WGCNA. **(A)** The collected genes were clustered and merged into different modules. **(B)** Module–trait associations. Each cell contains the corresponding correlation and p-value. The color bar indicates the correlation of each module with each sample.

Based on the differentially expressed gene analysis, we collected 2117 DEGs from three comparison groups and analysed them with the method of WGCNA. A total of 18 gene expression modules were collected (**Figure 6A**), and according to the module and trait correlation analysis, the coregulatory modules were identified by correlation coefficient > 0.5 and p value < 0.05 (**Figure 6B**).

**Figure 7:**
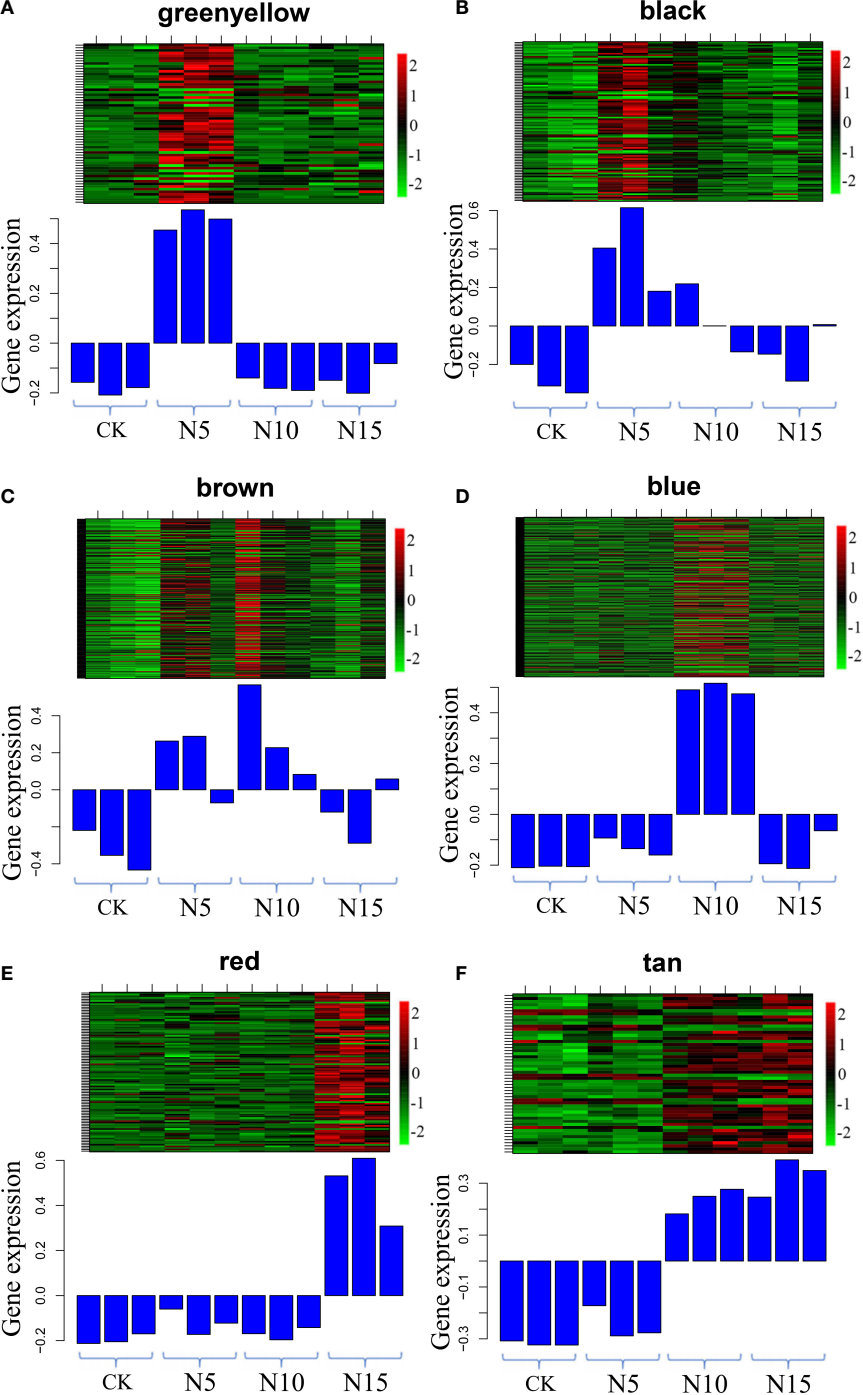
Gene expression profiles of selected modules. The color bar represent the relative expression, red denotes up-regulation and green denotes down-regulation. **(A-F)** The modules represented greenyellow, black, brown, blue, red and tan respectively.

**Figure 8:**
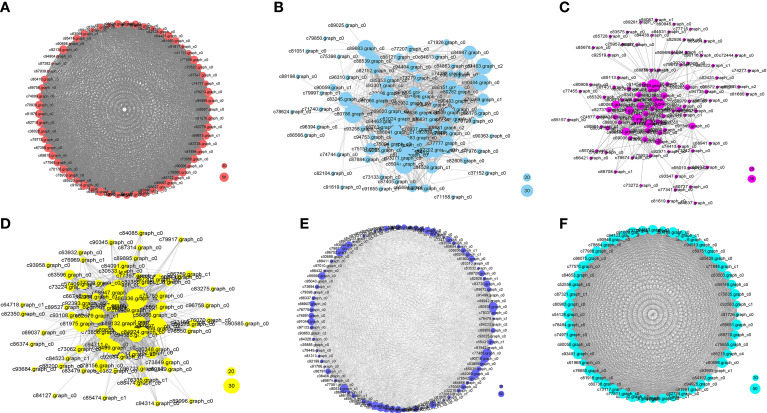
Network analysis of the collected modules. The bubble size indicates connect degree of each gene. **(A-F)** The networks represented greenyellow, black, brown, blue, red and tan respectively.

In the N5-vs.-CK comparison group, the green-yellow, salmon and black modules were collected. The expression profiles of genes in these three modules were depicted by heatmaps across all samples. A total of 61 DEGs were collected in the green-yellow module, and most of the upregulated genes were detected in the N5 group (**Figure 7A**). Based on the functional annotation, the collected genes in the green-yellow module included plant growth- and photosynthesis-related genes, such as *ARF* (c91123.graph_c0), auxin efflux carrier family protein (c90097.graph_c0) gene, sucrose synthase (*SuSy*, c90097.graph_c0) and chlorophyll a/b binding protein 2 (*Lhcb2*, c82134.graph_c0) genes, and secondary metabolism-related genes, which were enriched in ‘monoterpenoid biosynthesis’ and ‘flavonoid biosynthesis’ pathways, such as *CHI* (c78025.graph_c0), neomenthol dehydrogenase (*NMR*, c78860.graph_c0) gene (**Figure 8A**). In the black module, 100 DEGs were collected, and most of the upregulated genes were also detected in the N5 group (**Figure 7B**). In addition to *Lhcb3* (c88539.graph_c0) gene during photosynthesis and sugar metabolism related genes, other functional genes, such as the *POD* (c77777.graph_c0) gene, elongation factor (*EF*, c88198.graph_c0) gene, histone H3 (c69025.graph_c0) gene and E3 ubiquitin-protein ligase (*EL*, c71740.graph_c0) gene were collected in the network analysis (**Figure 8B**).

In the N10-vs.-CK comparison, the brown, blue, cyan and midnight blue modules were collected. A total of 212 DEGs were collected in the brown module, most of which were upregulated in the N10 group, while some of the genes were also upregulated in the N5 group (**Figure 7C**). The network analysis showed that some genes related to photosynthesis, such as the *psbP* (c78379.graph_c0) gene, *psaD* (c73194.graph_c0) gene and blue-light photoreceptor (c91454.graph_c0) gene, were upregulated in the N10 group. Furthermore, some enzyme synthesis and plant hormone related genes, such as *SOD* (c80060.graph_c0), *GST* (c81884.graph_c1), *POD* (c91380.graph_c0), and auxin-binding protein (c72444.graph_c0) gene were upregulated. In addition, some transcription factor genes were collected in this module, such as *MYB* (c84062.graph_c0, c92964.graph_c0), *bHLH* (c69458.graph_c0, c85512.graph_c0, c71271.graph_c0), *TCP* (c76145.graph_c0) and *ERF* (c76763.graph_c0) transcription factor genes, all of which were upregulated (**Figure 8C**). In the blue module, 275 DEGs were most of which were upregulated in the N10 group (**Figure 7D**). Except for some photosynthesis, plant hormone-related and transcription factor genes, such as *Lhcb2* (c81347.graph_c0) gene, *psaA* and *psaB* (c81180.graph_c0) gene, auxin responsive protein (c69869.graph_c0) gene, *ERF* (c68175.graph_c0), *SBP* (c84523.graph_c1) and *MYB* (c76609.graph_c0) transcription factor genes and other metabolism and functional genes include the nitronate monooxygenase (c82612.graph_c0) gene, *SuSy* (c81588.graph_c1) gene, *β-AS* (c91264.graph_c0), histone H3 (c57400.graph_c0) gene, histone H4 (c30533.graph_c0) gene and zinc finger protein (c93267.graph_c0, c91298.graph_c0) gene were collected in the network (**Figure 8D**).

In the N15-vs.-CK comparison group, four modules with red, pink, tan and lightcyan were collected. A total of 101 DEGs were collected in the red module, most of which were upregulated in the N15 group (**Figure 7E**). The network analysis of these DEGs showed that metabolism biosynthesis-related genes, such as the *SuSy* (c95043.graph_c0) gene and germacrene D synthase (c73694.graph_c1, c95043.graph_c0) gene, were collected. Furthermore, other functional genes included transcription factors and plant hormone-related genes, such as brassinosteroid insensitive 1-associated kinase 1 (*BRI1*, c86844.graph_c1) gene, PIN-FORMED (PIN) protein (*PIN*, c89411.graph_c0) gene, zinc finger protein (*COL3*, c78275.graph_c0) gene and *AP2* (c89411.graph_c0) gene were also collected (**Figure 8E**). In the tan module, 52 DEGs were most of which were upregulated in the N15 and N10 groups (**Figure 7F**). Many of the upregulated genes in the network, such as *POD* (c94513.graph_c0), *ERF* (c67768.graph_c0, c77988.graph_c0), *LHY* (c81968.graph_c0), zinc finger protein (*COL2*, c89792.graph_c1) gene and *SuSy* (c86075.graph_c0) gene, were identified (**Figure 8F**), some of which were also upregulated in the N10 groups. Secondary metabolism related genes, such *CAD* (c89681.graph_c0) gene was downregulated in both the N15 and N10 groups. The functional analysis of these collected modules shown that most of DEGs may involved in photosynthesis, plant growth and development and secondary metabolism process (**Table 2**). These collected genes may be vital for the regulation of the response in the N additional environment.

**Table 2:** Functional analysis of collected modules with network analysis.

Module names	Number of genes	GO terms	KEGG pahtways	Related genes
greenyellow	61	Photosynthesis, light harvesting in photosystem I	Monoterpenoid biosynthesis	*ARF*, *CHI*, *Lhcb2*, *MNR*, *SuSy*, etc.
		Regulation of ARF protein signal transduction	Isoflavonoid biosynthesis	
		Chloroplast organization	Phenylpropanoid biosynthesis	
		Developmental growth	Photosynthesis - antenna proteins	
			Flavonoid biosynthesis	
			Starch and sucrose metabolism	
black	100	Protein heterodimerization activity	Photosynthesis - antenna proteins	*EF*, *POD*, *Lhcb3*, *Lhcb4*, etc.
		Integral component of membrane	Phenylpropanoid biosynthesis	
		Photosynthesis, light harvesting in photosystem I		
brown	212	Photosystem II oxygen evolving complex	Photosynthesis	*PsbP*, *PsaD*, *CRY*, *EL*, etc.
		Superoxide dismutase activity	Peroxisome	
		Regulation of transcription		
		peroxidase activity		
		DNA-binding transcription factor activity		
		Antioxidant activity		
blue	275	Integral component of membrane	Sesquiterpenoid and triterpenoid biosynthesis	*EL*, *Lhcb2*, *ERF*, *SBP*, *psaA*, *psaB*, *SuSy*, *β-AS*, etc.
		Signal transduction	Starch and sucrose metabolism	
		Nitronate monooxygenase activity	Photosynthesis	
			Nitrogen metabolism	
red	101	Auxin-activated signaling pathway	Plant hormone signal transduction	*SuSy*, *BRI1*, *PIN*, *AP2*, *COL3*, etc.
		Zinc ion binding	Starch and sucrose metabolism	
		Terpene synthase activity	MAPK signaling pathway - plant	
			Sesquiterpenoid and triterpenoid biosynthesis	
			Circadian rhythm - plant	
tan	52	DNA-binding transcription factor activity	Plant hormone signal transduction	*POD*, *ERF*, *LHY*, *CAD*, *COL2*, etc.
		Starch biosynthetic process	Circadian rhythm - plant	
		Hydrogen peroxide catabolic process	Phenylpropanoid biosynthesis	

### Verification of gene expression by qRT-PCR

**Figure 9:**
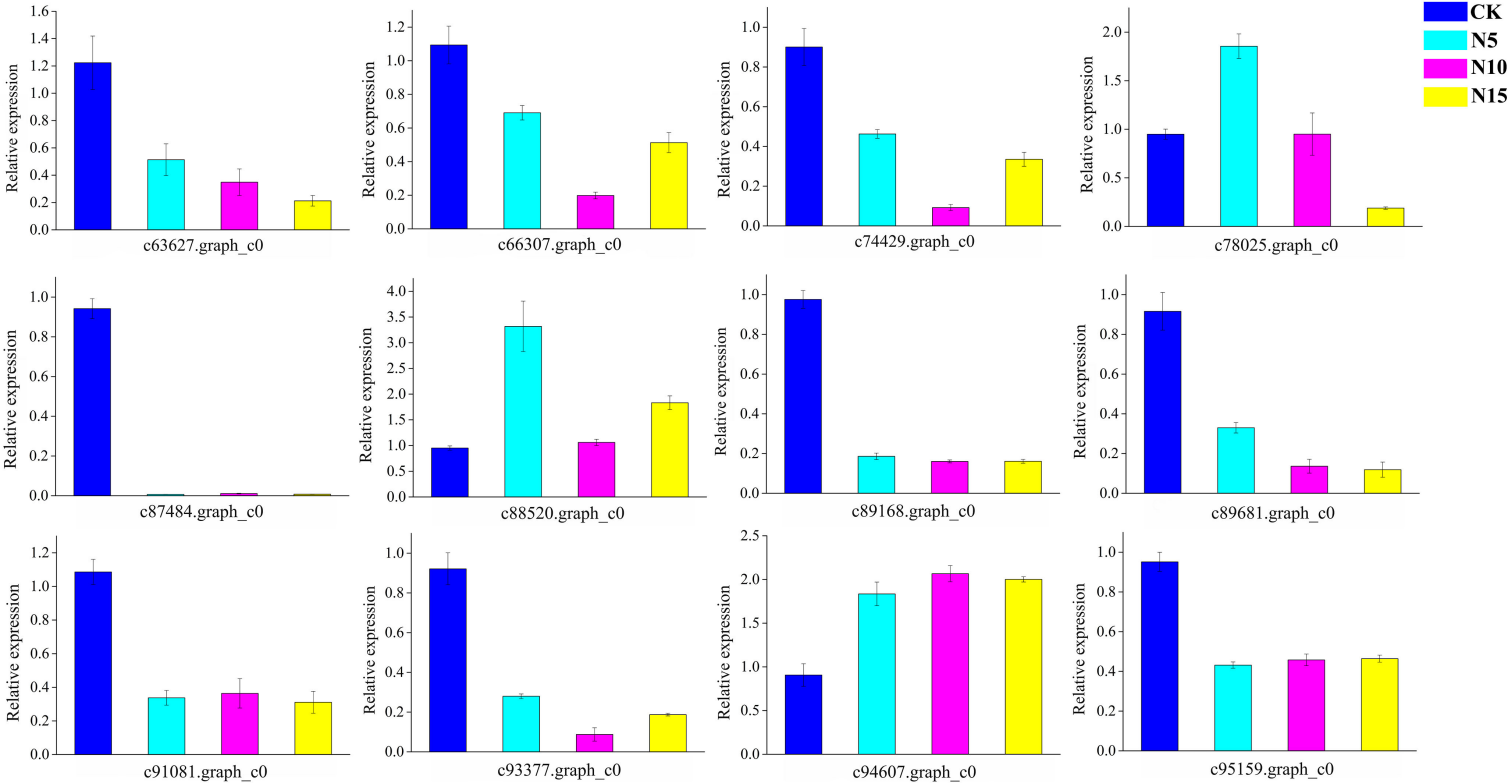
The qRT-PCR analyses of randomly selected genes in CK, N5, N10, and N15 group respectively.

We randomly selected 12 candidate genes for qRT-PCR to validate the gene expression results, which were obtained from RNA-seq (**Figure 9**). As a result of qRT-PCR, the relative expression of each selected gene was detected and was basically in agreement with the RNA-seq data. The primer sequences are listed in the **Supplementary Table S7**.

## Discussion

### Physiological responses of *S. canadensis* in the N addition environment

Physiological investigations are essential and effective means to evaluate the real influence of various environments (Hu et al., 2019). Physiological traits such as photosynthetic pigments, antioxidant enzymes, and soluble sugars have often been implicated in survival and ecological strategies in plants under different conditions (Mao et al., 2017). Plants, especially invasive plants, in different N-level environments exhibit various physiological and morphological responses (Wan et al., 2019; Huang et al., 2022; Sun et al., 2022). The increased N supply environment can induce a significant effect on resource use in plants (Wang et al., 2017; Wan et al., 2019); therefore, continuous N addition conditions will promote plant invasion (Liu et al., 2018a; Luo et al., 2020), such as facilitating invasive plants to synthesize more photosynthetic compounds in the leaves, which supports plant growth (Huang et al., 2022). The physiological analysis showed that Chl contents increased with elevated N levels, which may contribute to the photosynthetic capacity and growth of *S. canadensis*. The GO and KEGG pathway enrichment analyses also showed that many genes were related to the GO terms ‘photosynthesis’ and ‘photosystem I reaction center’ and the KEGG pathway ‘photosynthesis-antenna proteins’, which may be related to the Chl contents in response to the N addition environment. Sugars, as energy sources in plants, the metabolism of which can be regulated by N levels (Sun et al., 2019; Zhang et al., 2021), play regulatory roles in plant growth and development (Lastdrager et al., 2014; Ciereszko, 2018). In this study, the soluble sugar content was upaccumulated with the N levels and reached the highest in the N10 environment. This suggests that the N environment may have an effect on the metabolic process of sugars in *S. canadensis*. Gene expression pattern analysis has shown that the transcription of numerous sucrose and starch metabolism-related genes will change sugar content levels (Gao et al., 2018; Zhu et al., 2018; Shao et al., 2020). The KEGG pathway enrichment analysis found that 35 DEGs were enriched in the ‘starch and sucrose metabolism’ pathway, with 12 enriched in the N5-vs.-CK and N15-vs.-CK comparison groups and 22 DEGs in the N10-vs.-CK comparison groups. The collected functional DEGs might be affected by the N environment and play an essential role in sugar metabolism in *S. canadensis*. In addition, previous studies have shown that antioxidant enzyme activity in plants is altered in N supplementation and fertilizer environments (Liao et al., 2019; Wang et al., 2022). With the results of this study, all three enzyme activities were elevated with N supplementation levels. For instance, the increased activities of SOD, POD and CAT were significantly elevated in the N-supplemented environment. Based on the RNA-seq dataset, DEGs encoding these enzymes were identified among the comparison groups, which might contribute to the enhanced activity of these enzymes in *S. canadensis* under the N level environment. Therefore, we can reasonably infer that the activity of these enzymes might be highly regulated at the protein level. Further research into higher levels of regulation will expand our current understanding of how *S. canadensis* responds to the N-supplemented environment.

### Secondary metabolite synthesis in *S. canadensis* may be altered by the N environment

Plants respond to global changes by simulating special physiological and molecular responses and by modulating distinct metabolism in plant growth and development (Zandalinas et al., 2022). Therefore, plants can adapt to new energy requirements brought about by different climate and environmental scenarios (Dusenge et al., 2019; Fernie et al., 2020). In alien invasive plants, allelopathy is one of the important approaches for successful invasion. Thus, the production of allelopathic chemicals makes introduced invasive plants more competitive against local plants than natives. The allelopathic potential of *S. canadensis* has significantly affected the production of secondary metabolites, which contribute to its competitive abilities and its successful invasion (Wu et al., 2020a). Previous researchers found that simulated N deposition can reduce phenolic compound synthesis in plants (Sun et al., 2020). The mechanistic evidence of this phenomenon was driven by the effects on PAL, which acts as the rate-limiting enzyme for the generation of phenylpropanoids. Therefore, both *PAL* gene expression and PAL enzyme activity were markedly inhibited by simulated N deposition. Furthermore, other phenylpropanoid metabolism- and phenolic synthesis-related enzymes of chalcone synthase (CHS) and flavanone-3-hydroxylase (FHT) have also been reported (Deng et al., 2019). In this study, most DEGs involved in phenylpropanoid and flavonoid synthesis were collected and downregulated under N environmental conditions, such as the *PAL*, *CAD*, *CHS*, chalcone isomerase (*CHI*) and *FHT* genes, encoding enzymes that promote phenylpropanoid and flavonoid secondary metabolite accumulation.

In addition, based on the KEGG enrichment analysis, some terpenoid synthesis-related DEGs were also collected. Many of these DEGs enriched in the sesquiterpenoid and triterpenoid synthesis pathways were downregulated in the N addition environment, such as the squalene monooxygenase gene, nerolidol synthase gene, α-farnesene synthase gene and *β-AS* gene. In the monoterpenoid and diterpenoid pathways, except for some of the downregulated DEGs in the N addition environment, such as the 8-hydroxygeraniol dehydrogenase gene, neomenthol dehydrogenase gene, gibberellin 3β-dioxygenase gene, some DEGs were upregulated, such as the (E)-8-carboxylinalool synthase gene, *KS* gene and gibberellin 2beta-dioxygenase gene. The results suggest that the difference in secondary metabolism-related genes may especially respond to the N environment, which may give rise to the specific accumulation of secondary metabolites. It is thought that the ability of plants to synthesize large numbers of specialized secondary metabolites helps them adapt to changing environments (Panda et al., 2021). These results indicate that the N addition environment may alter the secondary metabolism process in *S. canadensis*, which would affect allelopathic abilities and change plant chemical defenses with climate change.

### Transcriptome analysis of the effects of the N addition response of *S. canadensis*

Plant invasion is a major component of global change that could affected by other components of global change (Liu et al., 2018b; Ren et al., 2020). It is predicted that plant invasions will further increase as the global environment continues to change (Liu et al., 2017). Environmental change can increase the dispersal rate and population growth of alien species and may promote their invasion range (Sandel and Dangremond, 2012; Liu et al., 2017). In this study, some DEGs were detected of *S. canadensis* among different N level environment, which may play an important role in responding to changes in N addition environment and promote to spread in new area.

Nitrogen is often the most limiting nutrient for plant growth in most ecosystems, which is very important for fast-growing plants, especially invasive plants (Uddin and Robinson, 2018; Zhang et al., 2022). Invasive plants have more advantages in N allocation than native plants, which will promote the response of invasive plants to the N environment (Hu et al., 2022; Oduor, 2022). To explore the response of *S. canadensis* under a N environment, transcriptome analysis was performed among different N deposition level environments. Previous studies suggested that N levels can significantly affect the growth of *S. canadensis* (Wan et al., 2018). The successful invasion of the exotic plant *S. canadensis* may be significantly affected by the N addition environment (Liu X. S. et al., 2022). In this study, most plant growth- and development-related genes were differentially expressed in the N environment comparison groups. Based on the essential functional GO terms related to the N metabolism process, plant growth and photosystem, such as ‘nitrate assimilation’, ‘reactive nitrogen species metabolic process’, ‘nitrogen cycle metabolic process’, ‘nitrogen utilization’, ‘photosystem I reaction center’, and ‘growth factor activity’. KEGG enrichment analysis showed that many DEGs with the function of plant development-related pathways were significantly enriched, such as plant hormone, sugar metabolism and circadian rhythm-related pathways. As a result of response of other invasive plants under N deposition environment (Huang et al., 2022), N supplement conditions may change the growth, energy metabolism rate and availability of N resources of *S. canadensis*.

Furthermore, according to the gene expression network analysis, in addition to the plant growth- and development-related DEGs, many regulator genes were collected in the different modules, such as histone genes, zinc finger protein genes, E3 ubiquitin-protein ligase genes and transcription factor genes. These DEGs may play regulatory roles in plant growth and development in an N-addition environment.

## Conclusion

In this study, we explored the effect of the N environment response of *S. canadensis* in physiology and transcriptome analysis. The physiological data showed that the photosynthetic pigment and soluble sugar contents were elevated by N addition. The antioxidant enzyme activities were altered in different N level environments. Transcriptome analysis showed that photosynthesis- and sugar metabolism process-related genes were also upregulated under N addition. In addition, secondary metabolism-related genes were affected in the N environment, and most of the phenol, flavonoid and sesquiterpenoid and triterpenoid metabolism-related DEGs were downregulated in the N environment. Most monoterpenoid- and diterpenoid-related DEGs were upregulated. As a result, many regulated genes were collected and may play vital roles in *S. canadensis* under N addition. Taken together, our work provides valuable information for the responses of *S. canadensis* to the N deposition environment, and the elevated N level environment may facilitate the fast growth and spread of invasive plant species.

## Data availability statement

The datasets presented in this study can be found in online repositories. The names of the repository/repositories and accession number(s) can be found in the article/**Supplementary Material**.

## Author contributions

MW designed the research, analyzed the data and wrote the manuscript. HL provided the methodology and revised the manuscript. YZ and MS performed the experiments. Investigation. BL and TZ participated in the design of the study. All authors contributed to the article and approved the submitted version.
